# DNA damage-induced cell cycle checkpoints involve both p53-dependent and -independent pathways: role of telomere repeat binding factor 2

**DOI:** 10.1054/bjoc.2001.2002

**Published:** 2001-09

**Authors:** S Narayan, A S Jaiswal, A S Multani, S Pathak

**Affiliations:** UF Shands Cancer Center and Anatomy and Cell Biology, College of Medicine, University of Florida, Gainesville, FL 32610; Departments of Cancer Biology and, Laboratory Medicine, The University of Texas MD Anderson Cancer Center, Houston, Texas 77030, USA

**Keywords:** DNA damage, *p53*, telomere, TRF1, TRF2, cell cycle arrest, apoptosis

## Abstract

Treatment of colon cancer cells with MNNG causes DNA damage with reduced telomeric signals in a p53-dependent manner, but increased cell cycle arrest in S-G_2_/M by both p53-dependent and independent mechanisms. Results also indicate that cellular levels of TRF2 may play a critical role in MNNG-induced cell cycle arrest and apoptosis of colon cancer cells. © 2001 Cancer Research Campaign http://www.bjcancer.com


*British Journal of Cancer* (2001) **85**(6), 898–901
© 2001 Cancer Research Campaign
doi: 10.1054/ bjoc.2001.2002, available online at http://www.idealibrary.com on IDEAL

Short Communication

### DNA damage-induced cell cycle checkpoints involve both p53-dependent and -independent pathways: role of telomere repeat binding factor 2

**S Narayan[1], AS Jaiswal[1], AS Multani[2] and S Pathak[2,3]**

[1]UF Shands Cancer Center and Anatomy and Cell Biology, College of Medicine, University of Florida, Gainesville, FL 32610; Departments of [2]Cancer Biology and [3]Laboratory Medicine, The University of Texas MD Anderson Cancer Center, Houston, Texas 77030, USA

**Summary** Treatment of colon cancer cells with MNNG causes DNA damage with reduced telomeric signals in a p53-dependent manner, but increased cell cycle arrest in S-$G_2$/M by both p53-dependent and independent mechanisms. Results also indicate that cellular levels of TRF2 may play a critical role in MNNG-induced cell cycle arrest and apoptosis of colon cancer cells. © 2001 Cancer Research Campaign http://www.bjcancer.com

**Keywords**: DNA damage; *p53*; telomere; TRF1; TRF2; cell cycle arrest; apoptosis

The tumour suppressor gene *p53* is frequently mutated in human colon cancer (for review see Kinzler and Vogelstein, 1996). Treatment of cells with DNA-damaging agents induces nuclear accumulation of p53, which then trans-activates the cell cycle- and/or apoptosis-related genes. A recently described mechanism that may trigger cell cycle arrest and apoptosis after DNA damage-response is telomere shortening of chromosomes (Ishibashi and Lippard, 1998; Multani et al, 1999, 2000; Pathak et al, 2000). Telomeres are specialized double-stranded 5´-TTAGGG-3´ DNA repeats of 2 to 30 kb that are present at the ends of chromosomes. The 3´ end of the telomere extends further for about 150 nt as a single-stranded repeat of 5´-TTAGGG-3´ that serves as a primer for telomerase in replication of the lagging-strand, which requires a short RNA template. In recent studies, it has been suggested that oxidative stress-mediated telomere shortening may induce p53-dependent cell cycle arrest (Saretzki et al, 1999). Furthermore, overexpression of wild-type p53 in human lung cancer cell lines is found to reduce telomeric signals and increase end-to-end telomeric association to trigger apoptosis (Mukhopadhyay et al, 1998).

### MATERIALS AND METHODS

### Maintenance and treatment of cells

Human colon cancer cell line HCT-116 with wild-type *p53* gene ($p53^{+/+}$) or with the *p53* gene-knocked out ($p53^{-/-}$), and SW480 cell line with a mutant *p53* gene (p53mut) were grown in McCoy's 5a medium with 10% fetal bovine serum. After cell cultures were 70% confluent, fresh medium without serum was added to each dish. Cells were further incubated for an additional 18 h and treated with N-methyl-N´-nitro-N-nitrosoguanidine (MNNG).

*Received 26 January 2001*
*Revised 22 May 2001*
*Accepted 24 May 2001*

*Correspondence to:* S Narayan

### Fluorescence in situ hybridization (FISH) and FACS analysis

The control and the treated colon cancer cell lines were harvested after indicated periods and processed for cytological preparations using the standard air-drying technique. A detailed FISH analysis protocol is described by Multani et al (1996). Propidium iodide staining of nuclei and their distribution into different phases of the cell cycle was determined by the use of a Becton–Dickinson FACScan flow cytometer (Bresnahan et al, 1996). The ranges for $G_0$/$G_1$, S, $G_2$/M and sub-$G_0$/$G_1$ phase cells were established on the basis of the corresponding DNA content of histograms.

### Telomerase activity and Western blot analysis

A polymerase chain reaction (PCR)-based telomerase activity detection kit TRAPEZE® (Intergen Company, Purchase, NY) was used in this study. The whole cell lysates were used for telomerase activity determined by TRAP (*T*elomeric *R*epeat *A*mplification *P*rotocol) as described by the manufacturer. The procedure for Western blot analysis was the same as described previously by Narayan and Jaiswal (1997). The antibodies used in these studies were p53(DO-1), p21(F-5), TRF1(C-19) and TRF2(N-20).

### RESULTS AND DISCUSSION

### The relationship between p53-dependent shortening of telomere length and apoptosis of colon cancer cells after treatment with MNNG

The isogenic HCT-116 human colon cancer cell lines with or without *p53* gene and SW480 cell line, with a missense mutations at amino acid residues 273 (arg to his) and 309 (pro to ser), were treated with MNNG for 50 h. Cells were processed for telomere signals by FISH analysis. Compared to the untreated cells, the telomere signals of MNNG-treated HCT-116($p53^{+/+}$) cells were significantly reduced (by 4-fold), while those of HCT-116($p53^{-/-}$) and SW480(p53mut) cells

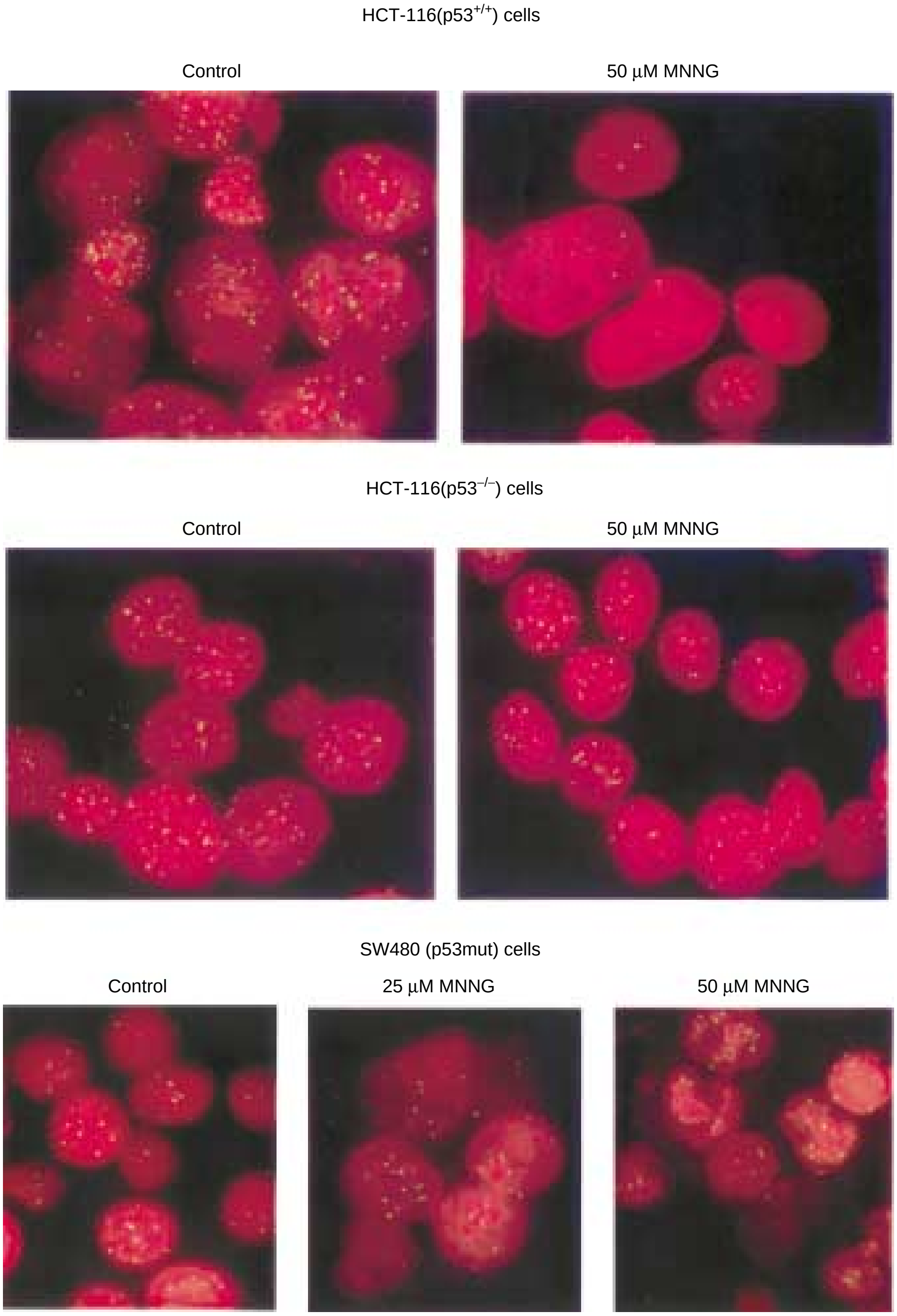

**Figure 1** MNNG-induced telomere signals in HCT-116($p53^{-/-}$), HCT-116($p53^{+/+}$), and SW480(p53mut) cell lines. (**A**) FISH analysis for telomere signals after treatment with MNNG for 50 h. (**B**) Western blot analysis of p53 and p21 protein levels after treatment with different concentrations of MNNG for 50 h. The yellow dots represent the signals for telomeric DNA. The photographs of the autoradiograms are representative of 3 independent experiments remained unchanged (Figure 1). This suggests that wild-type p53 is involved in MNNG-induced shortening of telomere length.

To test whether the telomere shortening and/or the wild-type p53 level is an important signal for cell cycle arrest and apoptosis, these cell lines were treated with different concentrations of MNNG for 50 h, and their cell cycle profile was measured by FACS analysis. The sub-$G_0/G_1$ peak of the FACSscan contained majority of the apoptotic cells (Hotz et al, 1994). We found MNNG-induced increase in the number of cells arrested in S-$G_2$/M phase with concomitant increase of the cells in sub-$G_0/G_1$ peak, which was irrespective to their p53 levels and telomere signals (Table 1).

### Telomerase activity of colon cancer cells is unchanged after treatment with MNNG

To examine whether telomerase activity of these cell lines changed after treatment with MNNG, a TRAP assay was performed. Results showed that the telomerase activity in HCT-116($p53^{+/+}$), HCT-116($p53^{-/-}$), and SW480(p53mut) cells remained unchanged

© 2001 Cancer Research Campaign

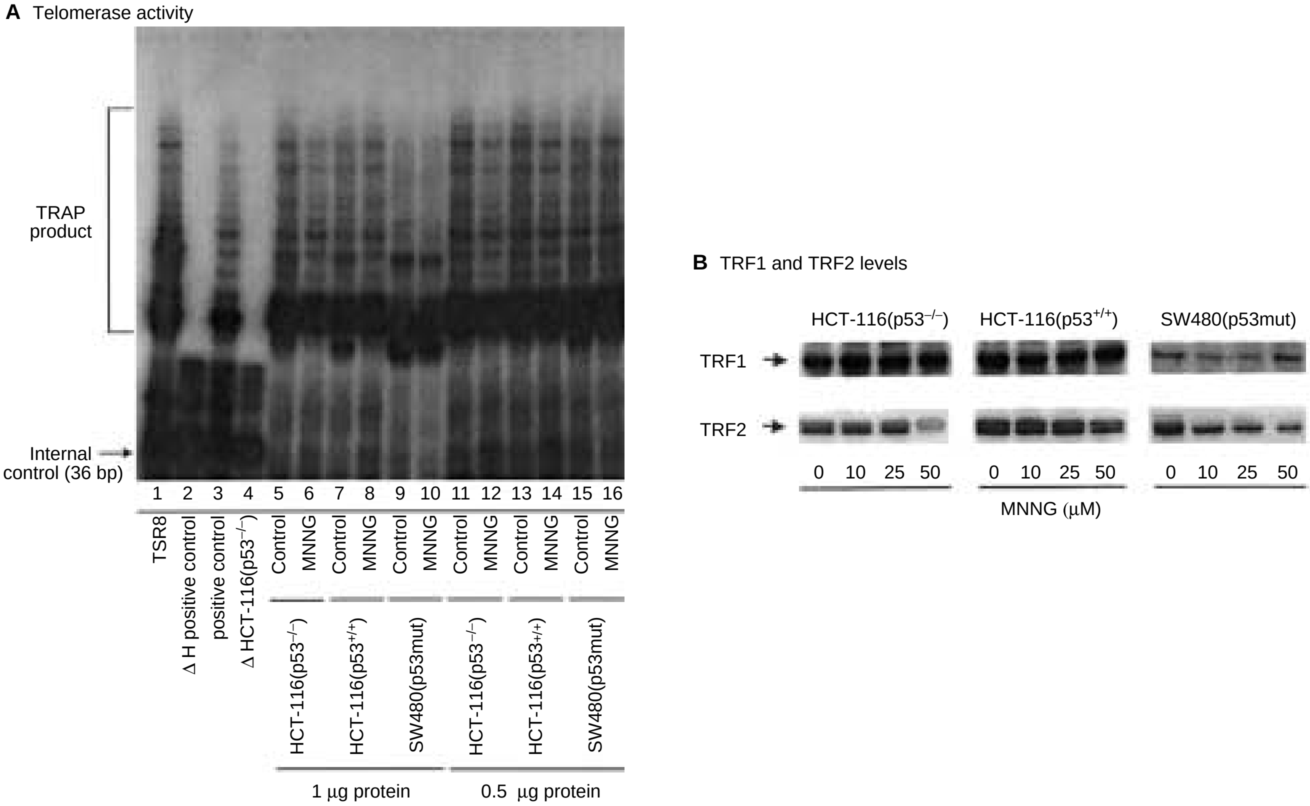

**Figure 2** (**A**) Telomerase activity. Cells were treated with 50 µM MNNG for 50 h. To determine the linear range of telomerase activity, the TRAP assay was performed with 2 protein concentrations. Bracketed area depicts the TRAP products and the arrow indicates the 36 bp internal control DNA. ΔH indicates the use of heat inactivated whole cell extract for the TRAP assay. TSR8 is an internal control oligonucleotide template with 8 telomeric repeats AG(GGTTAG)$_7$. (**B**) Western blot analysis for determining the levels of TRF1 and TRF2 after treatment of cells with different concentrations of MNNG for 50 h. Photographs of the autoradiograms are representative of 4 independent experiments

**Table 1** FACS analysis of cell cycle profile of colon cancer cell lines. Cells were treated with different concentrations of MNNG for 50 h and then analysed for cell cycle profile by flow cytometry. The distribution of cells in $G_0/G_1$, S, $G_2$/M, and sub-$G_1$ is shown

| | $G_0/G_1$ | S | $G_2$/M | sub-$G_0/G_1$ |
|---|---|---|---|---|
| MNNG (µM) | | HCT-116(p53$^{-/-}$) cells[a] | | |
| 0 | 80 ± 1.9 | 7 ± 0.1 | 7 ± 1.4 | 5 ± 1.3 |
| 5 | 75 ± 2.3 | 9 ± 1.5 | 12 ± 1.5* | 3 ± 1.0 |
| 10 | 64 ± 1.8* | 9 ± 1.2 | 18 ± 2.5* | 5 ± 1.0 |
| 25 | 16 ± 1.0* | 29 ± 4.1* | 41 ± 4.7* | 11 ± 1.8* |
| 50 | 38 ± 6.3* | 28 ± 4.0* | 16 ± 4.0 | 14 ± 2.7* |
| MNNG (µM) | | HCT-116(p53$^{+/+}$) cells[a] | | |
| 0 | 77 ± 1.8 | 7 ± 0.6 | 7 ± 1.2 | 8 ± 1.5 |
| 5 | 72 ± 3.8 | 8 ± 0.7 | 8 ± 2.0 | 10 ± 2.8 |
| 10 | 67 ± 3.0* | 10 ± 0.9* | 10 ± 1.3 | 11 ± 2.7* |
| 25 | 39 ± 3.5* | 12 ± 0.4* | 25 ± 2.4* | 22 ± 4.3* |
| 50 | 52 ± 2.9* | 10 ± 1.2* | 7 ± 1.0 | 29 ± 3.3* |
| MNNG (µM) | | SW480(p53mut) cells[b] | | |
| 0 | 67 | 12 | 7 | 11 |
| 5 | 34 | 20 (1.7) | 25 (3.4) | 17 (1.5) |
| 10 | 30 | 25 (2.1) | 27 (3.7) | 15 (1.3) |
| 25 | 27 | 28 (2.4) | 21 (2.9) | 20 (1.7) |
| 50 | 47 | 17 (1.5) | 9 (1.2) | 25 (2.2) |

* = Significantly different than control (unpaired *t*-test). [a] = Results are mean ± SE of 5–6 independent experiments. [b] = Results are mean of 2 independent experiments. Data shown in parenthesis are the fold increase as compared to control.

© 2001 Cancer Research Campaign after treatment with MNNG (Figure 2A). From these results, it appears that the level of p53, telomere shortening, and telomerase activity may not be determining factors for MNNG-induced cell cycle arrest and apoptosis.

### MNNG-induced expression of TRF2 level is critical for controlling cell cycle arrest in S-$G_2$/M

Chromosomal telomere length is controlled by specific 5´-*T*TAGGG-3´ *r*epeat binding *f*actors (TRFs) (Smogorzewska et al, 2000). In some cell types, TRF1 is identified as a suppressor of telomere elongation by a negative feedback mechanism that stabilizes telomere length. The increased level of TRF2 may protect telomere ends from degradation and ligation with an unknown mechanism yet to be discovered. However, it has been suggested that the loss of TRF2 causes a loss of 3´ G-rich overhangs at the telomere ends; resulting in DNA damage signals that may stimulate cell cycle arrest or apoptosis (for review see, Greider, 1999). In present studies, the level of TRF2 in HCT-116(p53$^{+/+}$), HCT-116(p53$^{-/-}$), and SW480(p53mut) cells significantly reduced and the level of TRF1 was unchanged after MNNG treatment (Figure 2B). This suggests that the reduced TRF2 levels may be critical for MNNG-induced cell cycle arrest and apoptosis.

TRF2 protein binds to the telomere end and facilitates formation of a t-loop. Once there is a loss of TRF2 protein, the t-loop structure is distorted and the telomere ends are free and easily accessible to endonuclease(s) that are involved in telomere shortening. Alternatively, without TRF2, the unprotected telomere ends may produce DNA damage responsive signals for cell cycle arrest and apoptosis without the shortening of the telomeres. Perhaps, a similar mechanism is operating in our experimental system. The HCT-116(p53$^{+/+}$) cells with wild-type p53 may respond to MNNG-induced S-$G_2$/M arrest by reducing the level of TRF2 protein, hence, distorting the t-loop structure and shortening the telomere ends later by unknown mechanisms. On the other hand, the MNNG-induced cell cycle arrest and apoptosis in HCT-116(p53$^{-/-}$) or SW480(p53mut) cells may arise due to loss of TRF2 levels resulting in the distortion of the t-loop structure. These results suggest that the maintenance of t-loop structure of the telomere is essential for cell survival.

### ACKNOWLEDGEMENTS

We are grateful to Dr Bert Vogelstein (The Johns Hopkins Oncology Center, The Johns Hopkins University, Baltimore, MD) for HCT-116(p53$^{-/-}$) and HCT-116(p53$^{+/+}$) cell lines, Dr Stratford May for critically reading the manuscript, Dr Virendra Kumar for technical assistance, and Nirupama Gupta for editorial comments. The FACS analysis was performed in the FCC Laboratory of the ICBR of the University of Florida. This work was supported by NIH grants CA77721 (to SN) and RRO4999 (to SP).

### REFERENCES

Bianchi A, Smith S, Chong L, Elias P and de Lange T (1997) TRF1 is a dimer and bends telomeric DNA. *EMBO J* **16**: 1785–1794

Bresnahan WA, Boldogh I, Thompson EA and Elbrecht T (1996) Human cytomegalovirus inhibits cellular DNA synthesis and arrests productively infected cells in late G1. *Virology* **224**: 150–160

Griffith JD, Comeau L, Rosenfield S, Stansel RM, Bianchi A, Moss H and de Lange T (1999) Mammalian telomeres end in a large duplex loop. *Cell* **97**: 503–514

Greider CW (1999) Telomeres do D-loop-T-loop. *Cell* **97**: 419–422

Hotz MA, Gong J, Traganos F and Darzynkiewicz Z (1994) Flow cytometric detection of apoptosis: comparison of the assays of in situ DNA degradation and chromatin changes. *Cytometry* **15**: 237–244

Ishibashi T and Lippard SJ (1998) Telomere loss in cells treated with cisplatin. *Proc Natl Acad Sci USA* **95**: 4219–4223

Kinzler KW and Vogelstein B (1996) Lessons from hereditary colorectal cancer. *Cell* **87**: 159–170

Mukhopadhyay T, Multani AS, Roth JA and Pathak S (1998) Reduced telomeric signals and increased telomeric associations in human lung cancer cell lines undergoing p53-mediated apoptosis. *Oncogene* **17**: 901–906

Multani AS, Hopwood VL and Pathak S (1996) A modified fluorescence in situ hybridization (FISH) technique. *Anticancer Res* **16**: 3435–3437

Multani AS, Li C, Ozen M, Imam AS, Wallace S and Pathak S (1999) Cell-killing by paclitaxel in a metastatic murine melanoma cell line is mediated by extensive telomere erosion with no decrease in telomerase activity. *Oncol Rep* **6**: 39–44

Multani AS, Ozen M, Narayan S, Kumar V, Chandra J, McConkey DJ, Newman RA and Pathak S (2000) Caspase-dependent apoptosis induced by telomere cleavage and TRF2 loss. *Neoplasia* **2**: 339–345

Narayan S and Jaiswal AS (1997) Activation of adenomatous polyposis coli (*APC*) gene expression by the DNA-alkylating agent N-methyl-N'-nitro-N-nitrosoguanidine requires p53. *J Biol Chem* **272**: 30619–30622

Pathak S, Multani AS, Narayan S, Kumar V and Newman RA (2000) Anvirzel, an extract of Nerium oleander, induces cell death in human but not murine cancer cells. *Anticancer Drugs* **11**: 455–463

Saretzki G, Sitte N, Merkel U, Wurm RE and von Zglinicki T (1999) Telomere shortening triggers a p53-dependent cell cycle arrest via accumulation of G-rich single stranded DNA fragments. *Oncogene* **18**: 5148–5158

Smogorzewska A, van Steensel B, Bianchi A, Oelmann S, Schaefer MR, Schnapp G and de Lange T (2000) Control of human telomere length by TRF1 and TRF2. *Mol Cell Biol* **20**: 1659–1668

*© 2001 Cancer Research Campaign* 

